# Survival, Growth and Condition of Freshwater Mussels: Effects of Municipal Wastewater Effluent

**DOI:** 10.1371/journal.pone.0128488

**Published:** 2015-06-04

**Authors:** Trey Nobles, Yixin Zhang

**Affiliations:** 1 Department of Biology, Texas State University, San Marcos, Texas, United States of America; 2 Department of Environmental Science, Xian Jiaotong-Liverpool University, Suzhou, China; Universite Pierre et Marie Curie, FRANCE

## Abstract

Freshwater mussels (Family Unionidae) are among the most imperiled group of organisms in the world, with nearly 65% of North American species considered endangered. Anthropogenic disturbances, including altered flow regimes, habitat alteration, and pollution, are the major driver of this group's decline. We investigated the effects of tertiary treated municipal wastewater effluent on survivorship, growth, and condition of freshwater mussels in experimental cages in a small Central Texas stream. We tested the effluent effects by measuring basic physical parameters of native three ridge mussels (*Amblema plicata*) and of non-native Asian clams (*Corbicula fluminea*), before and after 72-day exposure at four sites above and below a municipal wastewater treatment plant outfall. Survivorship and growth of the non-native Asian clams and growth and condition indices of the native three ridge mussels were significantly higher at the reference site above the outfall than in downstream sites. We attribute this reduction in fitness below the outfall to elevated nutrient and heavy metal concentrations, and the potential presence of other untested-for compounds commonly found in municipal effluent. These results, along with an absence of native mussels below the discharge, indicate a significant negative impact of wastewater effluent on both native and non-native mussels in the stream.

## Introduction

Surface freshwater ecosystems provide many services to human populations around the world, including the dilution and disposal of waste products [1]. In the United States, municipal wastewater treatment plants (WWTPs) are ubiquitous in urban and suburban areas. Although modern wastewater treatment technology has greatly reduced the amount of organic pollution, pathogens, and solids discharged into America’s streams and rivers, they still remain significant sources of inorganic nutrients, metals, pesticides, industrial chemicals, and pharmaceutical and personal care products (PPCPs) [2, 3]. The release of these substances in surface freshwaters can cause eutrophication and altered stream metabolisms [4, 5], disrupt reproductive and physiological processes in aquatic organisms [6], and influence community structure [7, 8].

Whole effluent testing (WET) is one approach often used when testing the toxicity of wastewater effluent on aquatic organisms. The approach of WET measures the toxicity of all known and unknown compounds in the effluent as well as any synergistic effects that may occur from combining multiple chemicals [9]. While most WET studies are conducted in laboratory settings, in-situ field trials often provide a more complete and relevant analysis of effluent toxicity in real-world settings [10], especially considering that the form, toxicity, and bioavailability of many toxins is dependent on water and sediment chemistry [11]. Active biomonitoring is one method of in-situ WET testing that involves collecting organisms from an unpolluted site and transplanting them to a test site to quantify their physical and biochemical responses to water quality [12]. Marine bivalves have been successfully used as active biomonitors for aquatic pollution for over 30 years [13], and more recently an increasing number of researchers have begun using freshwater mussels in biomonitoring programs [14].

Freshwater mussels offer several advantages over other organisms for in-situ WET testing [14, 15]. As benthic filter feeders, they are constantly exposed to dissolved and suspended materials in the water and sediment and ingest particulate matter through their filtering activity. Roditi et al. (2000) found that 77% of Ag, 78% of Cd, and 65% of Hg bioaccumulated in zebra mussels (*Dreissena polymorpha*) were obtained from food/nutrition [16]. Many metals were found adsorbed onto suspended particles [17] that would be filtered and processed by mussels. They are also more tolerant of handling stress than other commonly used aquatic organisms, and can be placed in smaller enclosures due to their sedentary lifestyle. Mussels also have a very high bioaccumulation rate and very low biotransformation potential for both organic and inorganic compounds, making them useful as long-term sentinels [10].

Ecologically, freshwater mussels are among the most threatened groups of aquatic organisms with 65% of North American species considered endangered [18, 19], which makes investigating anthropogenic impacts on this group particularly important [20]. Many species are relatively intolerant of elevated nutrient and toxin concentrations, especially during their larval and juvenile life stages [21, 22], and with only limited mobility as adults, sessile organisms probably cannot mitigate their exposure to pollutants by migration to areas with lower concentrations. Despite these facts, few studies have investigated how freshwater mussel populations are influenced by WWTPs, in terms of condition, growth, and survival. Those that have studied this have typically found reduced abundance and species richness downstream of discharges and increased mortality and reduced growth when exposed to effluent in caged field or laboratory trials [23, 24, 25]. None of these studies, however, have investigated the effects of tertiary-treated municipal wastewater in semi-arid streams that may become completely dominated by effluent during periods of drought.

In this study, we investigated the effects of tertiary-treated municipal wastewater effluent on transplanted native and non-native freshwater mussels in enclosures for 72 days in a small stream in central Texas. We measured survival, growth, and condition indices of the native threeridge mussel *Amblema plicata* (Say 1817), and survival and growth in the non-native Asian clam *Corbicula fluminea* (Muller 1774) after in-situ exposure to either one of three downstream sites below the WWTP outfall or the upstream reference site above the outfall. Based on the results of previously published studies and on preliminary water quality testing of the effluent plume at our study site, we predicted that mussels would have impaired survivorship, growth, and condition index immediately below the effluent discharge compared to the upstream reference site, and that the response in these variables would become more favorable with increasing distance downstream from the discharge.

## Materials and Methods

### Site description

We conducted our study in Wilbarger Creek (30°20'47.23"N, 97°32'56.74"W), a third order tributary of the Colorado River, which is located in eastern Travis County of Texas, with a watershed area of approximately 470 km^2^. Soils within the watershed are predominately dense clay, and land use is mainly pasture and cultivated agriculture, although the watershed also drains the rapidly growing towns of Pflugerville, Manor, and Elgin. Wilbarger Creek has a maximum-recorded discharge of 20,000 cubic feet per second (cfs) and is naturally a seasonally intermittent stream with zero discharge reported 29% of the time. Due to supplemental additions from WWTP discharges, however, many sections have become perennial and under drought conditions these sections may become completely dominated by undiluted wastewater effluent. Discharge at the nearest Lower Colorado River Authority (LCRA) Hydromet gauge approximately 24 km downstream of the study site ranged from 1 cfs to 8950 cfs with a median discharge of 5 cfs during the study period of February 24 through May 22, 2012. There are eight active municipal WWTPs that cumulatively discharge 1.95 million gallons of effluent per day (mgd) into Wilbarger Creek, but are permitted to discharge up to 12.4 mgd.

**Fig 1:**
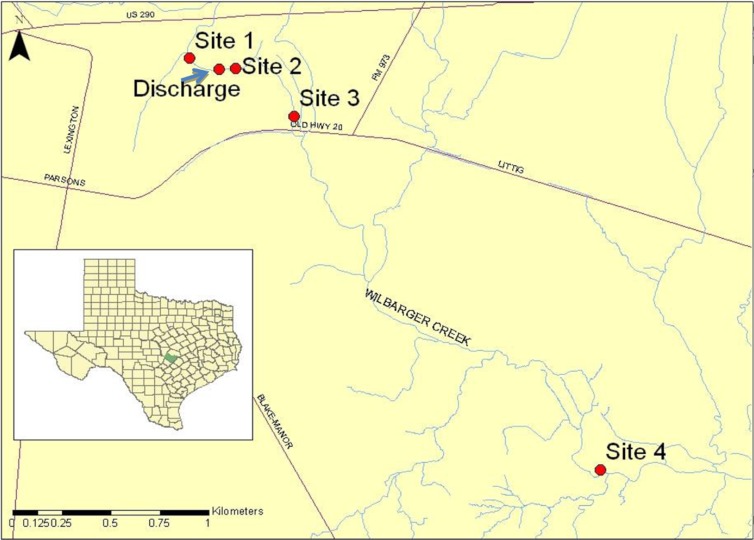
Map showing locations of the four study sites in relation to the Wilbarger wastewater treatment plant (WWTP) discharge (City of Manor, Travis County, Texas, 30°20'47.23"N, 97°32'56.74"W). Inset shows the location of Travis County (shown in green) in the state of Texas. Discharge indicates the discharge of the WWTP effluent.

In order to investigate the effects of municipal wastewater effluent on the Wilbarger Creek ecosystem, we chose four sites near the Wilbarger Creek Wastewater Treatment Facility (TPDES Permit No. WQ001290000l, 30°20'43.79"N 97°32'56.43"W) located in and operated by the city of Manor, TX. Current discharge is up to 0.5 mgd, with future permitted discharge up to 2 mgd (see Table 1 for effluent constituent limitations). We had initially planned on conducting a downstream gradient impact study with three 100 m long sites below the WWTP discharge (with the site farthest downstream as reference) as the area upstream of the discharge had been dry for the previous six months due to extreme drought in 2011. Heavy rains in January and February of 2012, however, restored flow upstream of the discharge and the reference site was selected above the discharge. In January 2012 (S1 Table), we conducted an initial water quality analysis at the discharge and at four sites up to 14.3 km downstream to map the effluent plume, and we used this data to determine our site locations (Table 2). Site 1, the reference site, was located approximately 160 meters upstream of the discharge, Site 2 approximately 50 meters below the discharge, Site 3 approximately 0.61 km downstream of the discharge, and Site 4 approximately 3.85 km downstream of the discharge (Fig 1). Sites 1, 2, and 3 were similar to each other and dominated by run habitat, whereas Site 4 was characterized by run and riffle habitat types (see Tables 3 and 4 for a full site description). In order to minimize the influence of different habitat types on the results of the study, we separated Site 4 into run and riffle habitats and only used run habitat data to compare results between sites. We performed stream habitat surveys by taking four transects at each site at the end of February approximately two weeks prior to beginning the in-stream enclosure studies. Flow data were collected using a Flow-Mate Model 2000 Water Current and Flow Meter (Flow-Tronic, Welkenraedt, Belgium), depth using a standard USGS wading staff, wetted and bank-full width using a 50-meter tape, and canopy cover using a convex forest densitometer. We collected water quality parameter data at each of the selected enclosure sites on three occasions between early March and mid-June. Dissolved oxygen, water temperature, conductivity, and pH were measured using a Hydro Tech Hydrolab MiniSonde 4a v2.0. Ammonia-nitrogen and nitrate and nitrite-nitrogen measurements (mg/L) were taken from 250 ml water samples and preserved with H_2_SO_4_. Total orthophosphorus measurements (mg/L) were taken from 250 ml water samples. Total suspended solids measurements were take from 500 ml water samples. *Escherichia coli* measurements (Most Probable Number/100 ml) were taken from 100 ml water samples and analyzed by the LCRA lab using Standard Method 9223B [26]. Following the completion of the study, we conducted a detailed water quality analysis of 37 common nutrients and pollutants in order to gain insights into possible drivers of the differences we found between sites. The LCRA National Environmental Laboratory Accreditation Conference (NELAC) certified laboratory located at 3505 Montopolis Drive in Austin, TX, performed analyses of all parameters not taken with the Hydrolab MiniSonde in accordance with the National Environmental Laboratory Accreditation Program (NELAP) standards and the International Standard Organization's (ISO) 17025 and 9001/9002 standards. All water samples were collected from the top one-third of the water column, being careful not to get surface debris or bottom sediments into the container. Water samples were held in the dark on ice during transport (approximately one hour travel time).

**Table 1:** Discharge limitations of effluent from the Wilbarger Wastewater Treatment Facility at 0.5 million gallons per day (MGD) discharge stage.

Effluent Characteristic	Daily Average (mg/L)	7-Day Avg. (mg/L)	Daily Max (mg/L)	Single Grab (mg/L)
Flow, MGD	Report	N/A	Report	N/A
Carbonaceous Biochemical Oxygen Demand (5-day)	5	10	20	30
Total Suspended Solids	5	10	20	30
Ammonia Nitrogen	2	5	10	15
Total Phosphorus	1	2	4	6
Total Dissolved Solids	Report	N/A	Report	N/A

**Table 2:** Major ions and basic water chemistry of the Edwards Aquifer water used to hold the study mussels in while at the lab.

Specific Conductance (μS/cm)	pH	Ca^2+^ (meq/L)	Mg^2+^ (meq/L)	Na^2+^ (meq/L)	K^+^ (meq/L)	Alkalinity (meq/L)	Cl^-^ (meq/L)	SO_4_^2-^ (meq/L)	Si (mmol/L)
596.00	7.00	4.43	1.43	0.53	0.04	5.19	0.58	0.55	0.19

**Table 3:** Preliminary water quality test results used to determine site locations.

Distance from outfall (km)	Conductivity (μS/cm)	pH	Dissolved Oxygen (mg/L)	Temperature (°C)	Total Suspended Solids (mg/L)	Total phosphorous (mg/L)	*E*. *coli* (Most Probable Number/100ml)	Ammonia (mg/L)	Nitrate (mg/L)
Effluent	1215	8	9.2	18	< 1	0.192	582	0.011	13.2
0.61	842	8	10.2	10.7	20.3	0.051	344	0.026	3.68
5.79	851	8.1	9.4	11.4	25	0.074	226	0.035	3.43
10.21	808	8.2	10.4	10.7	26.5	0.072	192	0.037	3.32
14.33	760	8.1	9.6	10.9	20.3	0.181	323	0.056	2.93

**Table 4:** Physical measurements and description of sites used in study.

Site	Distance downstream of discharge (km)	Habitat type	Substrate	Mean depth (m)^*a*^	Mean wetted width (m)^*a*^	Mean bankfull width (m)^*a*^	Mean canopy cover (%)^*a*^
1	-0.16	Run	silt	0.49 ± 0.09	6.8 ± 0.17	9.0 ± 0.41	64.5 ± 10.33
2	0.06	Run	silt	0.46 ± 0.14	6.0 ± 0.23	9.5 ± 0.65	76.5± 15.91
3	0.61	Run	silt	0.77 ± 0.19	5.4 ± 0.78	7.0 ± 0.71	0
4^*b*^	3.65	Run	silt/grvl/cobl	0.57 ± 0.19	5.4 ± 0.48	13.0 ± 2.01	77.6 ± 9.81

^*a*^ Value ± Standard Error.

^*b*^ Only run habitat data from Site 4 are included.

### Mussel community surveys

We collected freshwater mussels at each site by pulling 25 cm clam rakes with teeth spaced 2.5 cm apart (Eagle Claw Fishing Tackle Co., Denver, CO) through the substrate to a depth of approximately 10 cm through the entire 100 m reach. We identified collected mussels to species level using a key [27], measured total anterior to posterior shell length, and preserved them in 95% ethanol for conclusive identification in the lab. All collecting activities were conducted in publicly accessible areas under scientific collecting permit # SPR-1010-176 issued by Texas Parks and Wildlife Department, and no threatened or endangered species were disturbed.

### Experiment on mussel growth and condition indices

To study the effects of the wastewater effluent on freshwater mussels, we measured several physical parameters of native *Amblema plicata* (Say 1817) and non-native *Corbicula fluminea* (Muller 1774) both before and after in-situ exposure to the water at our four sites. *A*. *plicata* is a common and widespread mussel found throughout the eastern two-thirds of Texas. As previous mussel surveys in the study area showed a very low density of native mussels, we collected the *A*. *plicata* used in our study from a location on the Guadalupe River near Victoria, TX known to have a high density of mussels. Fifty six *A*. *plicata* of similar size (mean shell length 84.3 ± 3.53 mm) were collected by hand searching at the end of February 2012, placed into a large (89 L) aerated cooler filled with river water (20°C), and transported back to our lab (approximate drive time 2 hours). As in the mussel community survey, all collecting activities were conducted in publicly accessible areas under a scientific collecting permit issued by Texas Parks and Wildlife Department, and no threatened or endangered species were disturbed or involved in the present study. Since freshwater mussels are non-cephalopod invertebrates, Institutional Animal Care and Use Committee (IACUC) approval and regulation did not apply. The mussels were maintained in aerated river water and allowed to acclimate at room temperature (21°C) overnight. The following day, we removed approximately 15 L of river water from the cooler every hour for four hours and replaced it with artesian spring water, warmed to room temperature, from the Edward’s Aquifer formation that is piped into our lab (see Table 2).

Each mussel was then marked with an individually numbered tag (The Bee Works, Orillia, ON, Canada) affixed to the left valve with cyanoacrylate gel glue. We then measured length, width, and thickness using digital Vernier calipers (ThermoFisher Scientific, Waltham, MA, U.S.A.) and measured total wet mass using an Ohaus ScoutPro digital balance (Ohaus Corporation, Pine Brook, NJ, U.S.A.). We calculated an initial live mussel body condition index (BCI) as the whole wet mass of the mussel divided by shell length (BCI-wet) (S2 Table). This ratio is commonly used to measure growth and nutritive status in live bivalves [28].

We used juvenile *C*. *fluminea* to compare growth rates between sites, as they are known to grow up to 0.95 mm in length per week under warm water conditions [29] and adult unionid mussels are unlikely to exhibit measureable growth in length over the course of a short-term study. Two days prior to beginning the field portion of the study, we collected 80 juvenile *C*. *fluminea* (mean length ± Standard Error (SE)) 8.72 ± 0.05 mm, mean mass 0.15 ± 0.004 g) upstream of the WWTP discharge at Site 1 on Wilbarger Creek by sifting substrate through a 2 mm mesh sieve. The *C*. *fluminea* were transported back to the lab in buckets with stream water (travel time approximately 45 minutes), where they were randomly assigned to one of sixteen groups and held in individual containers of aerated stream water. We measured each individual’s length to two decimal places using digital Vernier calipers and the combined mass of all five mussels to four decimal places using a Mettler Toledo Classic Plus digital scale (Mettler Toledo LLC, Columbus, OH, U.S.A.). We used the mean length and mass of the mussels in each group to measure response to the effluent, as they were too small to mark individually for re-measurement (S3 Table).

We constructed our field experimental enclosures out of 27 × 38 × 43 cm plastic milk crates completely covered in 2.5 cm wire poultry mesh to prevent predation by fish or mammals. We attached 2 mm plastic canvas mesh to the bottom half of the crate’s sides using non-toxic hot glue, and filled the crates halfway with commercially available pea gravel for substrate. We constructed 8.5 × 8.5 × 8.5 cm cubes out of the 2 mm plastic canvas mesh to hold the *C*. *fluminea*, which were also filled halfway with pea gravel.

We placed four enclosures in the middle of the channel at each site approximately 2 m apart in a checkerboard pattern and anchored each cage with three steel concrete reinforcement rods. We placed one plastic mesh cube containing a group of five *C*. *fluminea* in each cage, and buried three randomly selected *A*. *plicata* halfway in the gravel substrate of each cage in their natural infaunal orientation. We checked the cages every two weeks to remove any accumulated debris and to ensure the cages had not been moved or lost by high flows.

We collected the enclosures on May 25 after 72 days of instream exposure and brought the mussels back to our lab for post-exposure measurements. We recorded the number of living *C*. *fluminea* and *A*. *plicata* in the cages and re-measured the same parameters as at the beginning of the study. We also dried the soft tissue of each *A*. *plicata* at 63°C for 48 hours to use in calculating a more accurate body condition index based on the proportion of the available internal shell cavity volume to actual soft tissue occupying that cavity (BCI-dry) [28]. The equation we used to calculate BCI-dry was dry soft tissue weight (g) × 1000 / internal shell cavity volume (ml), and is considered to be the most accurate measure of assessing the nutritive and stress status of bivalves [28].

### Statistical analysis

In order to determine the overall effect of site on growth and condition of *A*. *plicata*, we conducted a Multiple Analysis of Covariance (MANCOVA) test with our four measured parameters as dependent variables, site as factor variable, and pre-exposure whole wet mass as covariate [30]. For parameters that were measured both before and after the field study (wet mass and BCI-wet), we used the percent change in those parameters from pre-exposure to post-exposure in our analysis, and for parameters only measured post-exposure (dry tissue mass and BCI-vol), we used the data collected after retrieval of the enclosures. We followed the MANCOVA with one-way ANCOVA tests on each individual parameter, again with pre-exposure whole wet mass as covariate. When a significant difference was found, we conducted a Fisher’s LSD test to identify differences between the sites. We also conducted paired T-tests on the pre- and post-exposure measurements for each site to determine significant changes over time. Our experimental units for the statistical analyses of *A*. *plicata* were the sixteen cages, with measurements of individual mussels within each cage averaged to obtain an overall mean value for that cage. Statistical units for the *C*. *fluminea* were also each of the sixteen cages, with the combined whole wet mass of all five mussels per cage and average length of each mussel in each cage used in the statistical analyses for that cage. We conducted paired T-tests on pre- and post-exposure mass and length data within Sites 1 and 4, and also between the percent growths for Sites 1 and 4. Due to the high mortality of *C*. *fluminea* at Sites 2 and 3, statistical analyses for those parameters were not conducted at those sites. All analyses were conducted in SPSS with an alpha level of 0.05. Data were tested for normality and homogeneity of variance using Kolmogorov—Smirnoff and Levene’s tests, respectively.

## Results

### Water quality testing and mussel surveys

Preliminary water quality testing was conducted in January 2012, during a period of time when there were no upstream flows entering Wilbarger Creek in the immediate area above the WWTP outfall. This resulted in the water near the outfall becoming dominated by the effluent and different for several of our measured variables between the outfall and our next testing site 0.61 km downstream. Measurements were generally similar among the other tested locations (Table 4). Conductivity at the outfall measured 1215 μS/cm and ranged between 760 and 851 at the other sites. Total phosphorus measured 0.192 mg/L at the outfall, 0.051–0.072 mg/L between 0.61 and 10.21 km downstream, and increased back up to 0.181 at our testing site 14.33 km downstream. Nitrate was higher at the outfall at 13.2 mg/L than downstream sites, which ranged from 2.92–3.68 mg/L. *E*. *coli* bacteria counts were highest at the discharge with 582 mpn/100ml, and declined to 344 mpn/100ml at 0.61 km downstream and 323 mpn/100ml at 14.33 km downstream. Ammonia was lowest at the discharge with 0.011 mg/L, and increased to 0.056 mg/L at 14.33 km downstream. Temperature was 18.0°C at the discharge, and declined to 10.6–11.4°C downstream. Total suspended solids were below detectable limits (< 1 mg/L) at the discharge, and increased to 20.3–26.5 mg/L downstream. The pH values remained relatively consistent between all sites (8.0–8.2) and showed no obvious pattern. Dissolved oxygen ranged from 9.2 to 10.4 mg/L and also showed no obvious pattern.

The 72 day means of the basic suite of measured parameters showed little difference between the four enclosure sites, with the exception of nitrate/nitrite and orthophosphorus, which were higher at Site 4 than at the other three sites and *E*. *coli* which was higher at Sites 1 and 4 than at Sites 2 and 3 (Table 5). The effluent showed higher mean conductivity (1475 ± 86 μS/cm), ammonia (0.3 ± 0.3 mg/L), nitrate/nitrite (16.5 ± 3.4 mg/L), total phosphorus (0.8 ± 0.6 mg/L), and orthophosphorus (1.2 ± 0 mg/L) than at the enclosure sites.

**Table 5:** Maximum, minimum, and mean (± Standard Error) water quality parameters as measured throughout the study period (March 10—May 25).

Site (distance downstream from discharge)	Conductivity (μS/cm)	pH	Dissolved Oxygen (mg/L)	Temperature (°C)	Ammonia As Nitrogen (mg/L)	Nitrate/Nitrite As Nitrogen (mg/L)	Total Phosphorus (mg/L)	Orthophosphorus As Phosphorus (mg/L)	Total Suspended Solids (mg/L)	Chlorine (mg/L)	*E*. *Coli* (Most Probable No./100 mL)
Site 1 (-0.16 km)											
Maximum	1190	8.03	7.65	27.35	0.049	4.29	0.139	0.053	75.7	0.36	727
Minimum	877	7.75	5.15	19.51	0.008	0.024	0.083	0.004	31.4	0.1	131
Mean (± SE)	993 ± 71	7.9 ± 0.06	6.3 ± 0.6	23.4 ± 1.6	0.03 ± 0.008	1.8 ± 1.0	0.1 ± 0.01	0.02 ± 0.01	48.3 ± 9.8	0.2 ± 0.07	381.0 ± 129.3
Effluent (0 km)											
Maximum	1677	8.02	9.15	27.42	0.626	19.9	1.41	1.18	1	0.66	582
Minimum	1215	7.75	7.54	18.02	0.011	13.2	0.192	1.18	< 1	0.66	23
Mean (± SE)	1475 ± 86	7.8 ± 0.05	7.9 ± 0.3	24.1 ± 1.7	0.3 ± 0.3	16.5 ± 3.4	0.8 ± 0.6	1.2 ± 0	1.0 ± 0	0.7 ± 0	302.5 ± 279.5
Site 2 (0.05 km)											
Maximum	1093	8	7.83	24.55	0.084	4.05	0.261	0.148	56.5	0.1	345
Minimum	967	7.86	6.37	19.84	0.008	1.57	0.058	0.004	24.3	0.1	42
Mean (± SE)	1049 ± 41	7.9 ± 0.04	6.9 ± 0.4	22.7 ± 1.5	0.04 ± 0.02	2.6 ± 0.7	0.2 ± 0.07	0.1 ± 0.05	40.9 ± 9.3	0.1 ± 0	169.7 ± 90.7
Site 3 (0.6 km)											
Maximum	1063	8.04	10.17	25.33	0.111	3.68	0.179	0.071	69	0.1	344
Minimum	842	7.85	5.52	10.74	0.026	2	0.051	0.028	20.3	0.1	36
Mean (± SE)	988 ± 48	7.9 ± 0.04	6.1 ± 1.1	22.7 ± 3.2	0.06 ± 0.02	2.8 ± 0.4	0.1 ± 0.03	0.06 ± 0.01	45.8 ± 13.4	0.1 ± 0	179.0 ± 63.5
Site 4 (3.85 km)											
Maximum	1320	8.05	7.45	26.49	0.045	6.87	0.341	0.68	62.5	0.11	651
Minimum	978	7.63	4.14	19.24	0.026	2.79	0.062	0.028	11	0.1	30
Mean (± SE)	1108 ± 74	7.9 ± 0.09	5.8 ± 0.7	23.5 ± 1.6	0.04 ± 0.004	4.1 ± 0.4	0.2 ± 0.06	0.3 ± 0.1	33.9 ± 11.4	0.1 ± 0.003	304.3 ± 130.6

In the more detailed 37-parameter post-exposure analysis of the effluent and water from Sites 1 and 4 (Table 6), the effluent was higher in aluminum (20.2 μg/L), copper (5.5 μg/L), ammonia (0.626 mg/L), nitrate/nitrite (19.9 mg/L), orthophosphate (1.18 mg/L), total phosphorus (1.41 mg/L), potassium (19.9 mg/L), zinc (66.2 μg/L), and total in-situ chlorine (0.66 mg/L) than either of the two enclosure sites sampled. Between the two enclosure sites, Site 4 was higher than Site 1 in chloride (203 mg/L), copper (2.1 μg/L), ammonia (0.042 mg/L), nitrate/nitrite (6.87 mg/L), orthophosphate (0.226 mg/L), total phosphorus (0.341 mg/L), potassium (11.6 mg/L), and zinc (23.1 mg/L).

**Table 6:** Results of post-study water quality analysis of 37 nutrients and potential pollutants collected on 6-6-12 from Site 1 (0.06 km upstream of outfall), undiluted effluent, and Site 4 (3.85 km downstream of outfall).

Site (distance downstream from outfall)	Site 1 (-0.16 km)	Effluent discharge (0 km)	Site 4 (3.85 km)
Alkalinity, Total (As CaCO3) (mg/L CaCO3)	226	83	169
Aluminum (μg/L)	4.9	20.2	3.6
Arsenic (μg/L)	10.4	1.52	4.77
Barium (μg/L)	98.5	21.7	77.4
Boron (mg/L)	0.303	0.401	0.373
Bromide (mg/L)	1.08	0.49	0.604
Caffeine (μg/L)	0.475	0.474	0.478
Calcium (mg/L)	131	79.1	92.5
Camphor (μg/L)	0.475	0.474	0.478
Chloride (mg/L)	166	201	203
Copper (μg/L)	1.9	5.5	2.1
DEET (μg/L)	0.475	0.474	0.478
*E*. *coli* (Most Probable Number/100mL)	727	23	30
Fluoride (mg/L)	0.627	0.271	0.426
HHCB (μg/L)	0.475	0.474	0.478
Iron (mg/L)	0.02	0.02	0.02
Isophorone (μg/L)	0.475	0.474	0.478
Lead (μg/L)	0.4	0.4	0.4
Magnesium (mg/L)	10.7	15.9	11.3
Methyl Salicylate (μg/L)	0.951	0.949	0.955
Nitrogen, Ammonia (As N) (mg/L)	0.033	0.626	0.042
Nitrogen, Kjeldahl, Total (mg/L)	1.08	1.79	1.33
Nitrogen, Nitrate & Nitrite (mg/L)	0.024	19.9	6.87
Organic Carbon, Total (mg/L)	8.18	5.86	6.1
Phenol (μg/L)	0.951	0.949	0.955
Phosphorus, Orthophosphate (As P) (mg/L)	0.004	1.18	0.226
Phosphorus, Total (As P) (mg/L)	0.12	1.41	0.341
Potassium (mg/L)	5.94	19.9	11.6
Sodium (mg/L)	117	173	169
Strontium (μg/L)	1240	1130	1090
Sulfate (mg/L)	147	198	167
Suspended Solids (Residue, Non-Filterable) (mg/L)	38.8	1	11
Triethyl Citrate (μg/L)	0.475	0.474	0.478
Triphenyl Phosphate (μg/L)	0.475	0.474	0.478
Volatile Suspended Solids (mg/L)	7.14	1.02	2.4
Zinc (μg/L)	10.1	66.2	23.1
Total Chlorine (in-situ) (mg/L)	0.36	0.66	0.11

In mussel surveys before the experiment, we did not find any living mussels at Sites 1, 3, and 4. At Site 2 we collected two native pond mussels (*Ligumia subrostrata*).

### *Amblema plicata* growth and condition experiment

**Fig 2:**
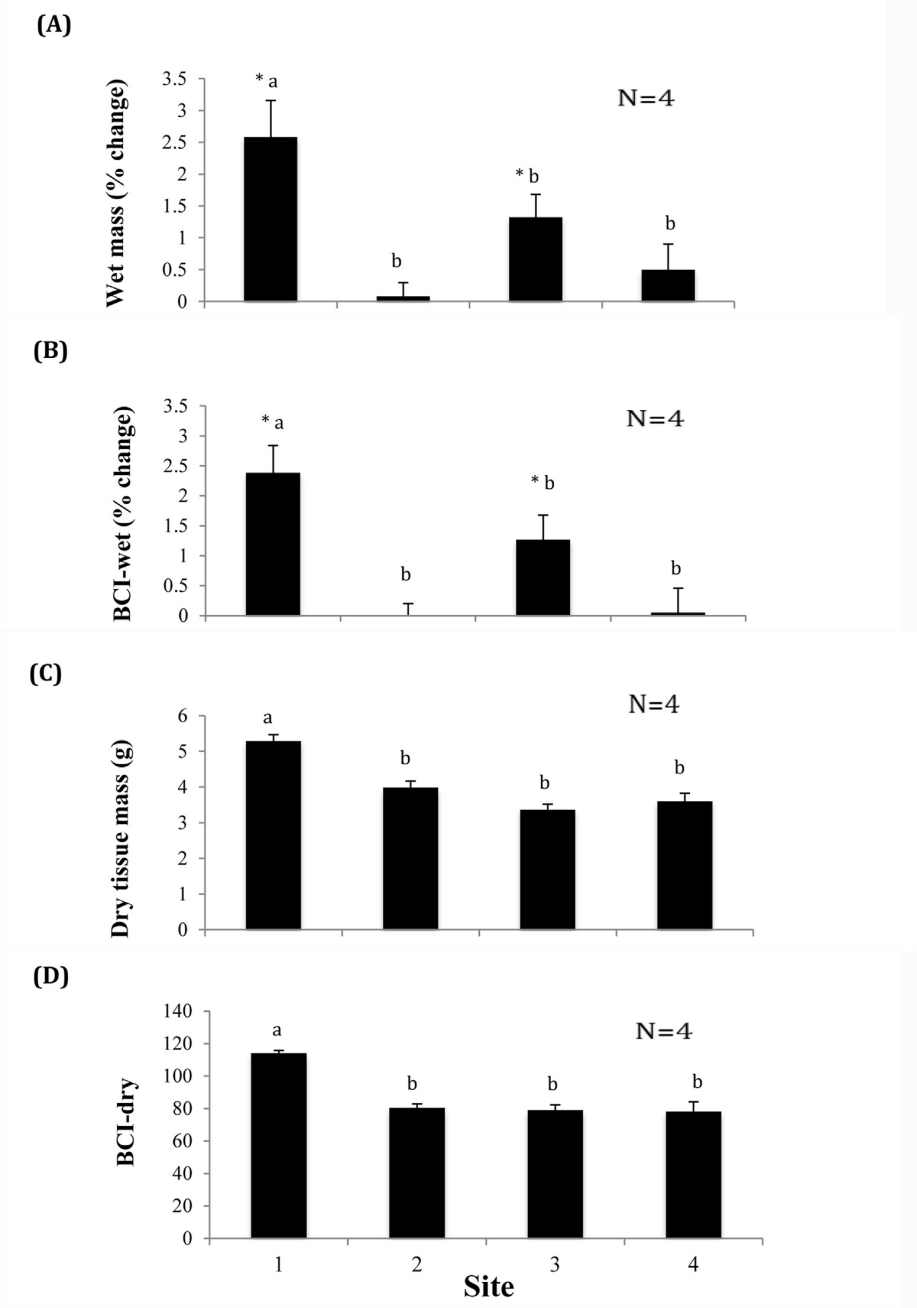
Physical responses of the native threeridge mussel (*Amblema plicata*) to effluent in the 72-day exposure experiment at four sites in Wilbarger Creek (Texas, USA). Error bars represent ± 1 standard error. Columns with the same letter were not statistically different from each other at p = 0.05. (A) and (B) represent change in pre-exposure (day 0) and post-exposure (day 72) wet mass and condition index respectively. An asterisk next to a column denotes significant (p<0.05) change in that parameter between day 0 and day 72. (C) and (D) represent post-exposure data only. Site 1, the reference site, was located upstream of the discharge, Site 2 just below the discharge, and Sites 3 and 4 downstream of the discharge.

Results of MANCOVA testing showed a significant overall difference between sites (F_21, 6.3_ = 3.858, p = 0.046) (Table 7). There were significant differences between sites for all four parameters: percent change in whole wet mass (ANCOVA: F_3, 11_ = 8.706, p = 0.003), percent change in BCI-wet (F_3, 11_ = 9.88, p = 0.002), BCI-dry (F_3, 11_ = 18.666, p < 0.001), and dry tissue mass (F_3, 11_ = 27.14, p < 0.001) (Table 7). Individuals of *A*. *plicata* above the outfall at Site 1 consistently showed the greatest increase in growth and better condition compared to those downstream. Percent increase in whole wet mass of each individual mussel was highest at Site 1 at 2.58 ± 0.58% (mean ± SE) and was significantly higher than the other three sites. Percent increase in whole wet mass was lowest immediately below the outfall at Site 2 at 0.08 ± 0.22% (Fig 2A). There was a significant increase in whole wet mass from pre- to post-exposure only at Sites 1 and 3 (Table 8). Sites 3 and 4 showed an increase of 1.32 ± 0.28% and 0.50 ± 0.40% respectively, and were not significantly different from Site 2. Percent increase in BCI-wet showed a similar pattern as whole wet mass. Site 1 showed the greatest increase at 2.38 ± 0.46%, and Site 2 the lowest with a slight decrease of -0.01 ± 0.42% (Fig 2B). Site 1 was again significantly higher than the downstream sites, which were all statistically similar to each other, and only Site 1 showed a significant increase in BCI-wet over time (Table 8). Post-exposure dry tissue mass was highest at Site 1 at 5.29 ± 0.18 g, and lowest at Site 3 at 3.37 ± 0.15 g (Fig 2C). Mean dry mass at Site 1 was significantly higher than at the downstream sites, which were not significantly different from each other. BCI-dry was highest at Site 1 with 113.96 ± 1.91 and lowest at Site 4 with 78.11 ± 6.04 (Fig 2D). Site 1 was significantly different from Sites 2, 3, and 4, which were all similar to each other.

**Table 7:** Results of MANCOVA and ANCOVA tests on *A*. *plicata* data.

	df (num, den)	F	Sig.
MANCOVA (all 4 parameters)	21, 6.3	3.858	0.046
Whole Wet Mass (% change)	3, 11	8.706	0.003
BCI-wet (% change)	3, 11	9.88	0.002
BCI-dry	3, 11	18.666	< 0.001
Dry tissue mass (g)	3, 11	27.14	< 0.001

Tests on whole wet mass and BCI-wet were performed on the percent change from pre- to post-exposure measurements. Tests on BCI-dry and dry tissue mass were performed on data collected post-mortem. MANCOVA was analyzed using all four measured parameters as dependent variables. Both MANCOVA and ANCOVA tests were run with the average pre- and post-exposure mussel whole wet mass as covariate.

**Table 8:** Mean mass and body condition index (BCI) values of pre- and post-exposure measurements of *A*. *plicata* and percent change (± Standard Error) for the parameters measured in each site.

Mass and body condition index (BCI)	Site 1 0.16 km above	Site 2 0.05 km below	Site 3 0.61 km below	Site 4 3.65 km below
Total mean wet mass, g (± SE)				
0 days	124.56 ± 4.45	131.67 ± 5.53	112.08 ± 3.02	122.76 ± 2.06
72 days	127.57 ± 4.14	131.73 ± 5.41	113.53 ± 2.77	123.39 ± 1.90
% Change	2.58 ± 0.58 *	0.08 ± 0.22	1.32 ± 0.28 *	0.50 ± 0.40
Mean BCI-wet (± SE)				
0 days	1.48 ± 0.04	1.52 ± 0.05	1.35 ± 0.04	1.46 ± 0.02
72 days	1.52 ± 0.04	1.52 ± 0.05	1.36 ± 0.04	1.46 ± 0.01
% Change	2.38 ± 0.46 **	-0.01 ± 0.42	1.26 ± 0.42	0.05 ± 0.41
Mean tissue dry mass, g (± SE)				
72 days	5.29 ± 0.18	3.98 ± 0.18	3.37 ± 0.15	3.60 ± 0.23
Mean BCI-dry (± SE)				
72 days	113.96 ± 1.91	80.39 ± 2.41	78.87 ± 3.52	78.11 ± 6.04

Mean tissue dry mass and mean BCI-dry values were only measured at day 72.

Significant results of t-tests comparing pre- and post-exposure data indicated by asterisks:

* Indicates p-value of < 0.05.

** indicates p-value of < 0.01.

### *Corbicula fluminea* survival and growth experiment

**Fig 3:**
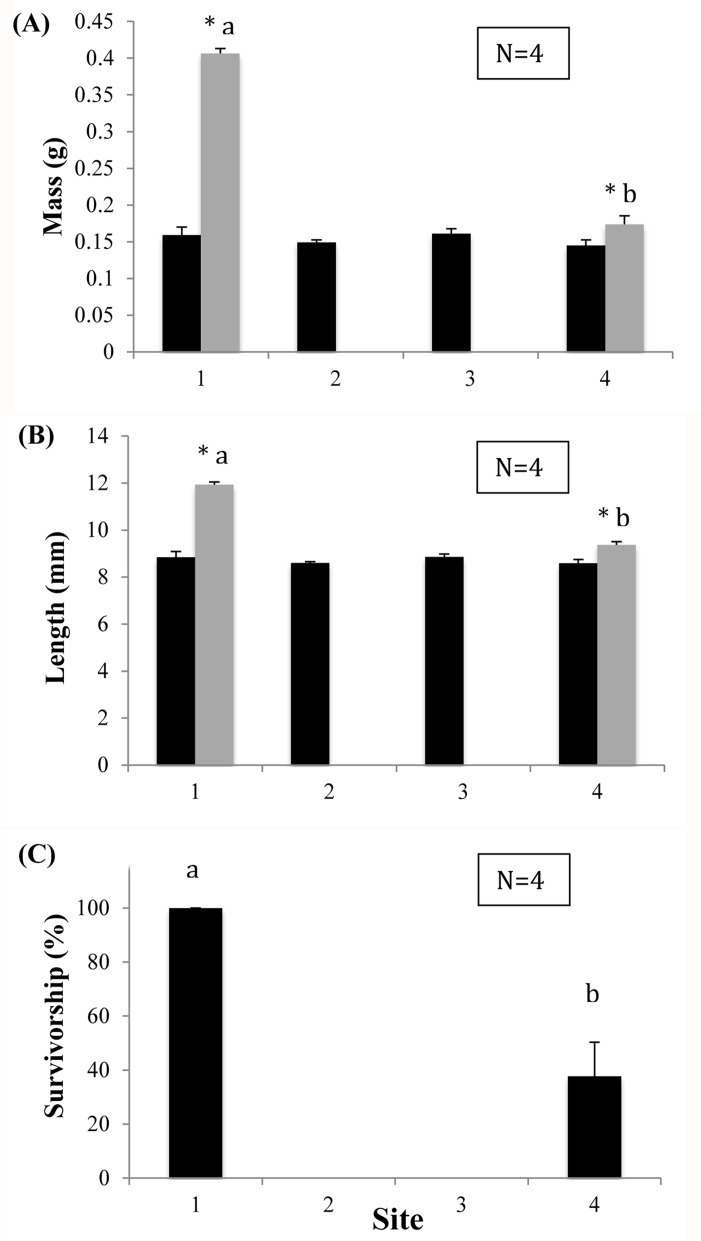
Growth and survival of the non-native Asian clam (*Corbicula fluminea*) after 72 days of exposure to effluent at four sites in Wilbarger Creek (Texas, USA). (A) Mean whole wet mass in grams at day 0 (black bars) and day 72 (gray bars) of the study; (B) mean total length in millimeters at day 0 (black bars) and day 72 (gray bars) of the study; (C) mean percent survivorship at day 72. Error bars represent ± 1 standard error. Columns with the same letter were not statistically different from each other at p = 0.05. An asterisk next to a column denotes significant change in that parameter between day 0 and day 72. Site 1, the reference site, was located upstream of the discharge, Site 2 just below the discharge, and Sites 3 and 4 downstream of the discharge.

Survival and growth of the *C*. *fluminea* differed greatly between upstream and downstream sites (Table 9). All individuals at Sites 2 and 3 died and we were unable to record any post-exposure data from them. Whole wet mass at Site 1 showed a significant increase of 159.79 ± 17.61% from an average of 0.16 ± 0.01 g/mussel pre-exposure to 0.41 ± 0.007 g/mussel post-exposure. Mussels at Site 4 showed a significant increase in whole wet mass of 23.48 ± 3.49% from 0.15 ± 0.008 g/mussel to 0.17 ± 0.01 g/mussel (Fig 3A). The increase in whole wet mass was significantly greater at Site 1 than at Site 4. Growth in length at Site 1 increased significantly by 35.22 ± 3.18% from 8.85 ± 0.13 mm/mussel to 11.94 ± 0.11 mm/mussel, and at Site 4 the mussels also increased significantly by 10.16 ± 1.12% from 8.59 ± 0.08 mm to 9.37 ± 0.14 mm (Fig 3B). The increase in length was also significantly greater at Site 1 than at Site 4. Survivorship ranged from 100 ± 0% above the outfall at Site 1 to 0 ± 0% below the outfall at Sites 2 and 3, with Site 4 showing intermediate survivorship of 37.78 ± 25.17% (Fig 3C). In addition to the five *C*. *fluminea* we placed in each enclosure at Site 1, we found a total of 33 additional juvenile *C*. *fluminea* in the cages upon retrieval. These new individuals were easily identifiable as recruits due to their smaller size compared to the five original ones we placed in the enclosures in February. No additional *C*. *fluminea* were found at any other site.

**Table 9:** Mean *C*. *fluminea* survivorship and pre- and post-exposure measurements and percent change (± Standard Error) for the physical parameters measured in our study for each site.

Mass, length and survivorship	Site 1 0.16 km above	Site 2 0.05 km below	Site 3 0.61 km below	Site 4 3.65 km below
Mean wet mass, g (± SE)				
0 days	0.16 ± 0.01	0.15 ± 0.00	0.16 ± 0.01	0.14 ± 0.01
72 days	0.41 ± 0.01	-	-	0.17 ± 0.00
% Change	158.79 ± 17.61 ***	-	-	23.48 ± 3.49 ***
Mean length, mm (± SE)				
0 days	8.85 ± 0.25	8.60 ± 0.06	8.86 ± 0.13	8.59 ± 0.16
72 days	11.94 ± 0.05	-	-	9.37 ± 0.14
% Change	35.22 ± 3.18 ***	-	-	10.16 ± 1.12 ***
Survivorship, % (± SE)				
72 days	100 ± 0	0 ± 0	0 ± 0	37.78 ± 25.17

Post-exposure data for Sites 2 and 3 are unavailable due to complete mortality of *C*. *fluminea* at those sites.

Significant results of t-tests comparing pre- and post-exposure data within sites indicated by asterisks:

*** Indicates p-value of < 0.001.

## Discussion

Results of this study generally agree with our initial hypothesis of decreased growth and increased mortality downstream of the effluent outfall. *A*. *plicata* at sites below the outfall exhibited significantly lower growth in whole wet mass, lower condition indices, and lower dry tissue mass than individuals at the reference site upstream of the outfall. Similarly, *C*. *fluminea* at Site 4 (3.85 km downstream of the outfall) showed significantly reduced growth and survivorship compared to those at Site 1 upstream of the outfall, with individuals at Sites 2 and 3 (0.06 and 0.61 km downstream respectively) exhibiting complete mortality. Although we did not see a consistent pattern of increased growth and condition in *A*. *plicata* with increasing distance from the outfall, we did see some survival and growth of the *C*. *fluminea* at our most downstream site. Results of this study suggest that the effluent from the Wilbarger WWTP could have a negative impact on the mussels and ecology of Wilbarger Creek downstream of its outfall for at least 3.85 km. Native *A*. *plicata* showed significantly lower growth in mass and condition indices below the outfall compared to the upstream reference site after 72 days exposure. The juvenile *C*. *fluminea* also exhibited significantly lower growth in length and mass and lower survival rates below the discharge, whereas all individuals at Site 1 survived and grew. Our mussel surveys found only two live mussels downstream of the effluent outfall, whereas we found one live and several dozen recently killed adult pond mussels in the dewatered streambed upstream of the outfall during our initial site visit in January.

Our results add to the growing body of knowledge suggesting the negative effects of wastewater effluent to bivalves. Horne and McIntosh (1979) found that mussel abundance declined from an average of 7.1 mussels/m^2^ above a secondary treated wastewater discharge on the Blanco River in Texas to 0.0 immediately below it, and density increased to only 0.2 mussels/m^2^ at 2 km downstream [23]. They also found zero survival of three species of native mussels (including *A*. *plicata*) after 28 days of exposure to diluted effluent in enclosures downstream of the outfall, with *C*. *fluminea* showing 50% survival downstream. They attributed this decline to elevated concentrations of ammonia and potassium in the diluted effluent (6.8 and 7.8 mg/L, respectively). Single sample ammonia concentrations in our study never exceeded 0.11 mg/L at any of our test sites, which is only slightly higher than the lowest reported acute LC_50_ concentration (the concentration of a chemical required to kill 50% of the test animals in a given time) for juvenile *C*. *fluminea* which are more sensitive to ammonia than native unionid mussels [31, 32]. Although ammonia toxicity studies using *A*. *plicata* have not been conducted, the concentrations of ammonia measured in our study are below the 0.3–0.7 mg/L range recommended by Augsburger et al. [31] as safe for continuous exposure to all life stages of freshwater mussels, including glochidia which are typically more sensitive to contaminants than adults [31]. Freshwater mussels are known to be sensitive to potassium [23, 33]. Potassium has been investigated as a possible biocidal agent to control Asian clam and zebra mussel (*Dreissena polymorpha*) infestations [34]. Imlay (1973) found potassium concentrations of 11 mg/L toxic to 90% of freshwater mussels tested between 36–52 days, and that 7 mg/L was lethal to two species after 8 months exposure [33]. Based on his findings and on an analysis of freshwater mussel distribution and potassium concentrations in 49 rivers, he recommended potassium levels should not exceed 4–10 mg/L for mussels. We measured potassium concentrations of 19.9 mg/L in the effluent and 11.6 mg/L 3.85 km downstream at Site 4, whereas concentrations upstream of the discharge at Site 1 were 5.9 mg/L. While these concentrations may explain the differences we found in growth of *A*. *plicata*, they are much lower than acute concentrations (120 mg/L) reported to induce shell gaping (a stress response) for *C*. *fluminea* [35].

Goudreau et al. (1993) also found greatly reduced densities of unionid mussels and *C*. *fluminea* below two WWTPs on the Clinch River in Virginia compared to upstream sites, but no differences in density above and below communities served by on-site septic systems [24]. Their study suggested that mussels had been eliminated below the WWTP discharges and glochidia from above the discharges were prevented from recolonizing downstream areas by some chemical pollutant in the effluent, most likely unionized ammonia and chlorine. Their water quality analyses revealed that total residual chlorine at sites just below the WWTPs regularly exceeded the 24 hour LC_50_ of 0.084 mg/L they established through laboratory testing. While instream ammonia levels only exceeded their assumed LC_50_ of 0.284 mg/L on one occasion at one site, they hypothesized that sublethal concentrations of both chlorine and ammonia could prevent the glochidia’s ability to successfully infest host fish and complete their life cycle. Gangloff et al. (2009) found similar differences in mussel abundance above and below a WWTP on Parkerson Mill Creek in Alabama, and also reported increased mortality (78%) and decreased condition of caged mussels downstream of the WWTP [25]. They, too, hypothesized that chlorine and/or other untested compounds were driving these differences (although not measured in their study, the WWTP being investigated had been frequently cited for high levels of chlorine). While ammonia concentrations at our sites only exceeded 0.284 mg/L in the undiluted effluent, total residual chlorine at all sites and all sampling dates was higher than the LC_50_ of 0.084 mg/L used by Goudreau et al. (1993) [24]. However, we found the highest mean concentration of chlorine (0.165 mg/L) *upstream* of the discharge at Site 1, where growth of both *A*. *plicata* and *C*. *fluminea* was highest and where we also noted the presence of many small juvenile *C*. *fluminea*, suggesting that chlorine from the Wilbarger WWTP is not significantly impacting mussels there.

Several studies have shown increased mortality to transplanted adult mussels below WWTP discharges [23, 25, 36] or to effluent in laboratory settings [36, 37, 38]. However, to our knowledge there have not been any studies published that were able to monitor extant mussel populations near newly built WWTPs, so we are unable to make any definitive conclusions about how mussel population structure changes when WWTPs begin discharging effluent. The presence of relatively healthy mussel populations above wastewater discharges and lack of mussels below them, as was the case in our study, indicates that recruitment of larvae is not occurring in areas of higher effluent concentrations [23–25, 36]. Mussel glochidia are known to be among the most sensitive aquatic organisms for many environmental contaminants commonly found in wastewater effluents [11, 31], and can be killed or immobilized at concentrations below that known to affect adults. Glochidia can exhibit an impaired valve-closure response to toxins, which can reduce their likelihood of successfully attaching to the gills of a host fish. Juvenile mussels often spend much of their time completely buried in the top layers of stream substrate and filter pore water [39], which can contain higher concentrations of ammonia and other toxins than surface water [11, 31].

Bringolf et al. (2010) found that female mussels altered their lure display behavior and released more nonviable glochidia than those in controls and that males released their spermatozeugmata prematurely in the presence of fluoxitene [40], which is the active ingredient in Prozac commonly found in municipal effluents [41]. Another study found that exposure to effluent had reduced the size of the seminiferous tubules in male *D*. *polymorpha*, reducing the sperm producing areas of the gonads and potentially reducing fecundity [42]. Estrogen-like compounds present in wastewater effluent have also been shown to induce feminization and skew sex ratios toward females in caged *E*. *complanata* [43]. Bayne et al. (1979) also found reduced fecundity and egg viability in the marine mussel *M*. *edulis* when placed under toxic stress [44]. These population-level impacts can have severe long-term consequences for freshwater mussels whose populations may already be impaired due to other environmental disturbances and ecological stress. The effluents of municipal WWTP can impose physiological stress, such as oxidative stress, on freshwater mussel [45]. In a short-term exposure experiment, Farcy et al. (2011) found that municipal effluents with a mixture of bacterial and chemical compounds had adverse physiological effects on the freshwater mussel *Elliptio complanata* [36].

## Conclusions

In this study, we have shown that growth, condition, and survival of both native and non-native mussel species may be significantly impaired by 72-day exposure downstream of a municipal wastewater treatment plant compared to an upstream reference site, at least up to 3.85 km downstream from the effluent discharge. The native *A*. *plicata* upstream of the effluent outfall increased significantly in total whole wet mass and condition index over a 72 day period and showed significantly higher growth in both metrics than mussels below the outfall. All non-native *C*. *fluminea* upstream of the outfall survived and increased their mean wet mass by 158% and their length by 35%, while 78% of individuals below the outfall died. Chronic exposure to municipal WWTP effluents with multiple contaminants negatively impacts freshwater mussel health and survival and may cause the decline of the freshwater mussel populations at local scale [46].

## Supporting Information

S1 Table Experimental Data for Native Three Ridge Mussels (*Amblema Plicata*). (PDF)

S2 Table Experimental Data for Non-Native Asian Clams (*Corbicula Fluminea*). (PDF)

S3 Table Water Quality Data of Studies Sites. (PDF)
