# Supplementary material for: Survival, Growth and Condition of Freshwater Mussels: Effects of Municipal Wastewater Effluent
Source: PLoS One. 2015 Jun 4;10(6):e0128488. doi: 10.1371/journal.pone.0128488 (PMC4456002; doi:10.1371/journal.pone.0128488)
Supplement: S1 Table — (PDF) [file pone.0128488.s001.pdf]

Water quality data of studies sites

11/04/2011 - Preliminary water quality sampling to determine effluent plume and sampling sites

Site	Distance from outfall (km)	Cond (µS/cm)	pH	DO (mg/L)	Temp (°C)	Turbidity (NTU)	Ortho P (mg/L)	Total Coliform (mpn/100ml)	E. coli (mpn/100ml)	Total Chlorine (mg/L)
Effluent	0	1155	7.9	8.7	21	7.85	0.42	1203	8	0.49
Wilbarger @ HWY 20	0.61	1209	7.7	6.3	11.9	10.55	0.36	>2419	11	0.07
Wilbarger @ Parsons	5.79	1349	7.9	7.3	10.8	14.16	0.52	1553	114.5	0.07
Wilbarger @ Jones	10.21	1406	8	7.6	12.3	17.14	0.09	1732	90.5	n/a
Wilbarger @ Bitting School Rd	14.33	1573	8.1	9.3	10.7	4.78	0.01	1732	410.6	0.03

01/30/2012 - Preliminary water quality sampling

Site	Distance from outfall (km)	Cond (µS/cm)	pH	DO (mg/L)	Temp(°C)	TSS (mg/L)	Total phosphorous (mg/L)	E. coli (mpn/100ml)	Ammonia (mg/L)	Nitrate (mg/L)
Wilbarger @ Outfall	0	1215	8.02	9.15	18.02	< 1	0.192	582	0.011	13.2
Wilbarger @ HWY 20	0.61	842	8.04	10.17	10.74	20.3	0.051	344	0.026	3.68
Wilbarger @ Parsons	5.79	851.5	8.11	9.35	11.43	25	0.074	226	0.035	3.43
Wilbarger @ Jones	10.21	808	8.15	10.4	10.66	26.5	0.072	192	0.037	3.32
Wilbarger @ Bitting School Rd	14.33	760	8.12	9.6	10.85	20.3	0.181	323	0.056	2.93

03/09/2012 - Routine water quality sampling after start of enclosure study

Site	Distance to outfall (km)	Cond (µS/cm)	pH	DO (mg/L)	Temp(°C)	Ammonia As N (mg/L)	Nitrate/Nitrite As N (mg/L)	Orthophosphorus As P (mg/L)	Total Suspended Solids (mg/L)	Chlorine (mg/L)	E. Coli Bacteria (MPN/100 mL)	Phosphorus As P (mg/L)
Site 1	-0.16	1000	8.03	7.65	19.51	0.49	4.29					
Effluent	0	1565	7.75	7.83	19.84	n/a	n/a	n/a	n/a	n/a	n/a	n/a
Site 2	0.05	1088	8	7.83	19.84	0.037	1.57	0.004	24.3	< 0.1	345	0.058
Site 3	0.61	1063	7.96	7.15	19.72	0.049	3.06	0.028	24.8	< 0.1	184	0.08
Site 4	3.85	1082	8.05	7.45	19.24	0.036	3.29	0.028	21.3	< 0.1	326	0.062

04/12/2012 - Routine water quality sampling after start of enclosure study

Site	Distance to outfall (km)	Cond (µS/cm)	pH	DO (mg/L)	Temp(°C)	Ammonia As N (mg/L)	Nitrate/Nitrite As N (mg/L)	Orthophosphorus As P (mg/L)	Total Suspended Solids (mg/L)	Chlorine (mg/L)	E Coli Bacteria (MPN/100 mL)	Phosphorus As P (mg/L)
Site 1	-0.16	903.8	7.88	5.15	22.48	0.027	2.38	0.008	75.7	< 0.1	416	0.139
Effluent	0	1677	7.92	8.4	23.74	n/a	n/a	n/a	n/a	n/a	n/a	n/a
Site 2	0.05	1093	7.86	6.37	23.74	0.084	2.27	0.133	56.5	< 0.1	42	0.261
Site 3	0.61	989	7.87	5.7	23.07	0.111	2.26	0.068	69	< 0.1	36	0.179
Site 4	3.85	1050	7.88	6	22.84	0.026	2.79	0.68	62.5	< 0.1	210	0.174

05/23/2012 - Routine water quality sampling after start of enclosure study

Site	Distance to outfall (km)	Cond (µS/cm)	pH	DO (mg/L)	Temp(°C)	Ammonia As N (mg/L)	Nitrate/Nitrite As N (mg/L)	Orthophosphorus As P (mg/L)	Total Suspended Solids (mg/L)	Chlorine (mg/L)	E Coli Bacteria (MPN/100 mL)	Phosphorus As P (mg/L)
Site 1	-0.16	876.8	7.96	6.83	24.41	0.008	0.518	0.013	48	< 0.1	131	0.083
Effluent	0	1294	7.87	7.54	25.2	n/a	n/a	n/a	n/a	n/a	n/a	n/a
Site 2	0.05	967.1	7.95	6.63	24.55	0.008	4.05	0.148	42	< 0.1	122	0.254
Site 3	0.61	913.1	7.85	5.52	25.33	0.44	2	0.071	68.9	< 0.1	152	0.176
Site 4	3.85	977.8	7.93	5.63	25.57	0.045	3.28	0.129	40.8	< 0.1	651	0.177

06/06/2012 - Post-study detailed water quality analysis of selected sites

Site	Site 1	Effluent	Site 4
Distance to outfall (km)	-0.16	0	3.85
Cond (µS/cm)	1190	1363	1320

pH		7.75	7.75	7.63
DO (mg/L)		5.58	7.72	4.14
Temp(°C)		27.35	27.42	26.49
Flow (cfs)		0	0.3	0.43
Alkalinity, Total (As CaCO3)	mg/L CaCO3	226	83	169
Aluminum	µg/L	4.9	20.2	3.6
Arsenic	µg/L	10.4	1.52	4.77
Barium	µg/L	98.5	21.7	77.4
Boron	mg/L	0.303	0.401	0.373
Bromide	mg/L	1.08	0.49	0.604
Caffeine	µg/L	0.475	0.474	0.478
Calcium	mg/L	131	79.1	92.5
Camphor	µg/L	0.475	0.474	0.478
Chloride	mg/L	166	201	203
Copper	µg/L	1.9	5.5	2.1
DEET	µg/L	0.475	0.474	0.478
Ecoli	MPN/100mL	727	23	30
Fluoride	mg/L	0.627	0.271	0.426
HHCB	µg/L	0.475	0.474	0.478
Iron	mg/L	0.02	0.02	0.02
Isophorone	µg/L	0.475	0.474	0.478
Lead	µg/L	0.4	0.4	0.4
Magnesium	mg/L	10.7	15.9	11.3
Methyl Salicylate	µg/L	0.951	0.949	0.955
Nitrogen, Ammonia (As N)	mg/L	0.033	0.626	0.042
Nitrogen, Kjeldahl, Total	mg/L	1.08	1.79	1.33
Nitrogen, Nitrate & Nitrite	mg/L	0.024	19.9	6.87
Organic Carbon, Total	mg/L	8.18	5.86	6.1
Phenol	µg/L	0.951	0.949	0.955
Phosphorus, Orthophosphate (As P)	mg/L	0.004	1.18	0.226
Phosphorus, Total (As P)	mg/L	0.12	1.41	0.341
Potassium	mg/L	5.94	19.9	11.6

Sodium	mg/L	117	173	169
Strontium	µg/L	1240	1130	1090
Sulfate	mg/L	147	198	167
Suspended Solids (Residue, Non-Filterable)	mg/L	38.8	1	11
Triethyl Citrate	µg/L	0.475	0.474	0.478
Triphenyl Phosphate	µg/L	0.475	0.474	0.478
Volatile Suspended Solids	mg/L	7.14	1.02	2.4
Zinc	µg/L	10.1	66.2	23.1
Total Chlorine (in-situ, COA)	mg/L	0.36	0.66	0.11
