# Supplementary material for: Survival, Growth and Condition of Freshwater Mussels: Effects of Municipal Wastewater Effluent
Source: PLoS One. 2015 Jun 4;10(6):e0128488. doi: 10.1371/journal.pone.0128488 (PMC4456002; doi:10.1371/journal.pone.0128488)
Supplement: S2 Table — (PDF) [file pone.0128488.s002.pdf]

Experimental data for native three ridge mussels (*Amblema plicata*)

			Pre-Exposure Measurements					Post-Exposure Measurements							
Cage	Site	Individual Number	Length (mm)	Width (mm)	Thickness (mm)	Tot. Wet Mass (g)	BCI-Wet (Total Wet Mass/Length)	Length (mm)	Width (mm)	Thickness (mm)	Tot. Wet Mass (g)	BCI-Wet (Total Wet Mass/Length)	Tissue Dry Mass (g)	Shell Cavity Volume (ml)	BCI-Dry (Tissue Dry Mass*1000/Shell Cavity Volume)
1	1	3	82.94	64.69	35.82	127.80	1.54	83.25	65.06	35.79	130.70	1.57	5.10	50	102.00
1	1	10	80.04	62.16	35.41	112.60	1.41	80.88	63.19	35.64	114.70	1.42	4.80	41	117.07
1	1	37	79.68	60.02	32.04	94.20	1.18	79.69	60.97	32.46	101.90	1.28	4.40	38	115.79
2	1	9	77.43	60.37	32.72	101.00	1.30	77.59	60.35	32.61	103.00	1.33	4.30	40	107.50
2	1	33	89.12	67.06	34.80	132.00	1.48	89.11	67.55	34.93	133.70	1.50	5.30	50	106.00
2	1	38	89.79	66.92	36.79	146.90	1.64	89.81	66.74	36.77	149.50	1.66	6.80	54	125.93
3	1	6	83.57	66.12	32.96	112.30	1.34	83.85	67.01	33.14	115.60	1.38	4.60	41	112.20
3	1	12	87.98	67.55	35.77	146.40	1.66	88.58	67.41	35.70	148.20	1.67	6.50	55	118.18
3	1	24	84.77	66.27	36.16	135.20	1.59	84.68	66.82	36.48	141.10	1.67	5.10	49	104.08
4	1	39	79.04	59.60	35.01	104.10	1.32	79.01	60.46	34.48	107.00	1.35	4.30	44	97.73
4	1	47	84.90	64.68	37.88	137.20	1.62	84.71	64.93	37.91	139.70	1.65	5.30	46	115.22
4	1	50	84.77	64.27	37.90	145.00	1.71	84.83	64.33	37.82	145.70	1.72	7.00	48	145.83
5	2	25	84.81	67.56	35.59	129.50	1.53	84.95	67.73	35.18	129.70	1.53	3.90	50	78.00
5	2	27	88.73	67.90	38.97	151.60	1.71	89.00	66.55	38.60	151.10	1.70	5.30	60	88.33
5	2	40	81.18	61.08	36.15	115.80	1.43	81.28	61.14	36.00	117.30	1.44	3.70	45	82.22
6	2	22	89.26	66.00	36.25	136.50	1.53	89.40	65.97	35.86	136.20	1.52	4.50	50	90.00
6	2	32	89.15	67.12	35.00	137.50	1.54	89.40	67.05	34.53	138.00	1.54	4.30	51	84.31
6	2	55	86.69	65.02	38.31	136.90	1.58	86.47	64.70	38.20	138.10	1.60	4.00	50	80.00
7	2	20	88.85	67.61	35.83	146.60	1.65	88.98	68.18	35.95	145.20	1.63	3.90	52	75.00
7	2	46	85.86	65.66	37.64	139.60	1.63	85.87	65.61	37.58	139.10	1.62	3.60	52	69.23
7	2	48	90.79	65.17	35.84	137.90	1.52	90.87	65.37	35.49	137.40	1.51	4.00	52	76.92

8	2	2	82.73	62.54	32.34	108.70	1.31	82.23	63.03	31.77	108.70	1.32	3.00	43	69.77
8	2	44	87.65	67.34	33.58	121.90	1.39	87.27	67.70	33.36	122.10	1.40	4.00	44	90.91
8	2	52	84.40	62.22	35.04	117.50	1.39	85.37	62.60	34.94	117.90	1.38	3.60	45	80.00
9	3	18	80.97	63.45	33.41	109.90	1.36	81.47	63.71	33.18	110.50	1.36	3.40	34	100.00
9	3	41	86.09	64.50	36.21	132.90	1.54	85.97	64.43	36.03	132.80	1.54	4.20	61	68.85
9	3	43	83.65	62.03	33.64	110.90	1.33	84.00	62.11	33.27	111.50	1.33	3.70	40	92.50
10	3	8	84.06	63.72	33.52	118.60	1.41	84.30	63.41	33.55	119.90	1.42	3.30	36	91.67
10	3	13	79.30	59.66	28.14	82.10	1.04	79.98	61.22	28.56	83.70	1.05	3.00	36	83.33
10	3	42	85.88	62.36	34.61	110.60	1.29	85.78	62.83	35.07	113.80	1.33	3.20	45	71.11
11	3	14	82.45	62.63	35.85	122.20	1.48	82.72	63.31	35.84	124.80	1.51	3.40	46	73.91
11	3	28	85.41	64.55	34.56	118.90	1.39	86.00	65.02	34.97	121.60	1.41	3.60	50	72.00
11	3	53	78.92	63.04	34.07	102.90	1.30	77.69	62.03	33.69	103.60	1.33	3.30	42	78.57
12	3	31	83.27	61.85	34.81	115.20	1.38	82.90	61.87	34.66	115.80	1.40	3.00	45	66.67
12	3	49	86.58	64.48	35.33	126.80	1.46	86.51	64.46	34.75	128.30	1.48	3.90	47	82.98
12	3	51	79.73	63.45	31.49	94.00	1.18	79.62	63.43	31.41	96.00	1.21	2.40	37	64.86
13	4	11	85.80	65.75	35.73	131.50	1.53	86.43	65.88	35.11	133.50	1.54	3.70	48	77.08
13	4	17	78.63	60.15	33.10	98.20	1.25	78.64	60.45	32.62	98.20	1.25	2.30	40	57.50
13	4	21	88.39	65.98	35.89	137.50	1.56	88.14	66.18	35.48	138.20	1.57	3.40	52	65.38
14	4	1	87.08	67.29	34.53	118.90	1.37	87.34	67.15	34.63	118.40	1.36	4.10	50	82.00
14	4	4	82.13	65.10	33.42	116.30	1.42	82.66	66.09	32.39	114.10	1.38	3.30	50	66.00
14	4	23	83.57	63.66	39.15	131.30	1.57	83.92	63.59	37.97	132.40	1.58	4.20	53	79.25
15	4	5	82.39	65.61	34.85	122.30	1.48	83.18	65.75	34.45	122.50	1.47	3.50	44	79.55
15	4	35	84.70	64.44	32.88	124.30	1.47	85.00	64.45	32.94	127.40	1.50	3.50	42	83.33
15	4	56	79.76	61.37	33.71	108.10	1.36	80.06	61.90	33.52	109.90	1.37	2.90	47	61.70
16	4	15	83.09	63.20	35.48	120.30	1.45	83.71	63.76	35.38	120.90	1.44	3.60	44	81.82
16	4	16	86.97	68.86	34.40	139.60	1.61	88.01	69.55	34.01	139.70	1.59	4.70	38	123.68
16	4	54	84.82	64.61	35.62	124.80	1.47	84.81	64.53	35.86	125.50	1.48	4.00	50	80.00
