# Supplementary material for: Survival, Growth and Condition of Freshwater Mussels: Effects of Municipal Wastewater Effluent
Source: PLoS One. 2015 Jun 4;10(6):e0128488. doi: 10.1371/journal.pone.0128488 (PMC4456002; doi:10.1371/journal.pone.0128488)
Supplement: S3 Table — (PDF) [file pone.0128488.s003.pdf]

Experimental data for non-native Asian clams (*Corbicula fluminea*)

Pre-Exposure

		Individual Lengths							
Cage #	Site	Mussel 1	Mussel 2	Mussel 3	Mussel 4	Mussel 5	Total Mass	Mean Mass	Mean Length
1	1	9.11	8.93	8	8.92	8.63	0.773	0.1546	8.718
2	1	8.87	8.11	9.21	8.69	8.26	0.744	0.1487	8.628
3	1	8.23	8.16	8.24	8.58	9.11	0.712	0.1425	8.464
4	1	9.68	9.63	9.15	9.83	9.57	0.956	0.1912	9.572
5	2	9.25	8.72	8.25	9.12	8.09	0.757	0.1514	8.686
6	2	8.36	8.81	8.97	9.22	8.21	0.788	0.1576	8.714
7	2	8.59	8.44	8.22	8.51	8.61	0.711	0.1422	8.474
8	2	8.64	8.76	8.22	8.65	8.42	0.725	0.145	8.538
9	3	9.13	8.22	9.51	8.42	8.91	0.795	0.159	8.838
10	3	8.63	8.42	9.31	8.98	9.09	0.807	0.1614	8.886
11	3	9.33	8.67	9.8	8.87	9.15	0.893	0.1786	9.164
12	3	8.42	8.56	8.18	9.03	8.55	0.723	0.1447	8.548
13	4	8.08	8.17	8.04	8.14	8.32	0.623	0.1246	8.15
14	4	8.81	8.59	8.74	9.02	8.95	0.785	0.157	8.822
15	4	9.1	8.55	8.68	8.84	9.05	0.784	0.1568	8.844
16	4	8.87	8.57	8.54	8.54	8.14	0.707	0.1413	8.532

Post-Exposure

		Individual Lengths							
Cage #	Site	Mussel 1	Mussel 2	Mussel 3	Mussel 4	Mussel 5	Total Mass	Avg. Mass	Avg. Length
1	1	12.51	12.52	11.47	12.1	11.25	2.047	0.4094	11.97
2	1	11.66	12.48	12.2	11.35	10.86	1.953	0.3906	11.84
3	1	12.08	12.25	11.79	12.19	11.97	2.111	0.4222	11.883
4	1	11.67	11.57	12.51	11.9	12.62	2.02	0.404	12.055
5	2	D	D	D	M	M			
6	2	D	D	D	D	M			
7	2	D	D	D	D	D			
8	2	D	D	D	D	D			
9	3	D	D	D	D	M			
10	3	D	D	D	D	D			
11	3	D	D	D	D	M			
12	3	D	D	D	D	M			
13	4	9.09							
14	4	D	D	D	D	D			
15	4	9.56	9.6	9.48	D	D	0.589	0.1963	9.5467
16	4	9.47	D	D	D	D	0.165	0.165	9.47

D=Dead

M=Missing
